# Morphometric measurements of systemic atherosclerosis and visceral fat: Evidence from an autopsy study

**DOI:** 10.1371/journal.pone.0186630

**Published:** 2017-10-16

**Authors:** Aline Nishizawa, Claudia K. Suemoto, Daniela S. Farias-Itao, Fernanda M. Campos, Karen C. S. Silva, Marcio S. Bittencourt, Lea T. Grinberg, Renata E. P. Leite, Renata E. L. Ferretti-Rebustini, Jose M. Farfel, Wilson Jacob-Filho, Carlos A. Pasqualucci

**Affiliations:** 1 Laboratory of Cardiovascular Pathology (LIM-22), Department of Pathology, University of Sao Paulo Medical School, Sao Paulo, Brazil; 2 Pathophysiology in Aging Lab/Brazilian Aging Brain Study Group (LIM-22), University of Sao Paulo Medical School, Sao Paulo, Brazil; 3 Discipline of Geriatrics, University of Sao Paulo Medical School, Sao Paulo, Brazil; 4 Division of Internal Medicine, University Hospital and State of São Paulo Cancer Institute (ICESP), University of São Paulo, Sao Paulo, Brazil; 5 Preventive Medicine Center, Hospital Israelita Albert Einstein and School of Medicine, Faculdade Israelita de Ciência da Saúde Albert Einstein, São Paulo, Brazil; 6 Department of Neurology, Memory and Aging Center, University of California, San Francisco, United States of America; 7 Department of Pathology, University of Sao Paulo Medical School, Sao Paulo, Brazil; 8 Department of Medical Surgical Nursing, University of São Paulo Nursing School, Sao Paulo, Brazil; The Ohio State University, UNITED STATES

## Abstract

**Background:**

Morphometric measurements of systemic atherosclerosis and direct quantification of visceral fat are only possible using materials from autopsy studies. However, the few autopsy studies that have investigated the association of visceral fat with atherosclerosis had small sample sizes and focused on coronary arteries of young or middle-aged White subjects. We aimed to investigate the association of pericardial fat (PF) and abdominal visceral fat (AVF) with atherosclerosis in the aorta, coronary, carotid, and cerebral arteries in a large autopsy study.

**Materials and methods:**

We evaluated deceased subjects aged 30 years or above. We dissected and weighted the PF and the AVF and evaluated the atherosclerotic burden in the aorta, as well as the carotid, coronary, and cerebral arteries using morphometric measurements. We also investigated the interaction of PF and AVF with age regarding the atherosclerotic burden.

**Results:**

The mean age of the 240 included subjects was 64.8±15.3 years, and 63% was male. Greater PF was associated with a higher degree of aortic atherosclerosis after adjusting for confounding variables (coefficient = 4.39, 95% CI = 0.83; 7.94, p = 0.02). Greater AVF was associated with a higher coronary stenosis index (coefficient = 1.49, 95% CI = 0.15; 2.83, p = 0.03) and a greater number of coronary plaques (coefficient = 0.71, 95% CI = 0.24; 1.19, p = 0.003). We did not find an association of PF or AVF with carotid or cerebral atherosclerotic burden. We found a significant interaction of AVF (coefficient = -0.08; 95% CI = -0.14; -0.02, p = 0.009) and PF (coefficient = -0.87, 95% CI = -1.70; -0.04, p = 0.04) with age regarding carotid artery atherosclerotic burden.

**Conclusions:**

Greater AVF was associated with greater atherosclerotic burden and extent in coronary arteries, while greater PF correlated with a higher degree of atherosclerosis in the aorta.

## Introduction

Since 1980, the prevalence of obesity has more than doubled worldwide. In 2014, 39% of adults were overweight and 13% were obese across the globe [1]. Obesity may be related to atherosclerosis by a complex process that may involve a chronic inflammatory state, insulin resistance, dyslipidemia, and hypertension [2]. Previous epidemiological studies showed an association of coronary artery atherosclerosis with epicardial [3], pericardial (PF) [4–6], and abdominal visceral fat (AVF) [7–9]. PF consists of epicardial and paracardial fat, which is located between the visceral pericardium and the myocardium, and outside of the parietal pericardium, respectively [10]. The association between subclinical atherosclerosis as measured by carotid artery intima-media thickness (CIMT) and AVF [11] or epicardial fat [12] has also been described. Aortic atherosclerosis is associated with epicardial fat thickness, but PF was not evaluated [13]. Cerebral artery plaque volume is associated with AVF [14]. Despite such evidence, all of these studies used imaging methods [3–6, 11–15], which quantified visceral fat and atherosclerosis through indirect measurements.

Autopsy studies are the gold standard for evaluating the association of visceral fat with atherosclerosis [16], allowing the direct measurement of atherosclerosis and the exact quantification of visceral fat [17]. However, the few autopsy studies that have investigated the association between visceral fat and atherosclerosis restricted their analyses to coronary arteries [7, 8, 18–20]. Moreover, the majority of the studies evaluated White young and middle-aged adults [7, 8, 20, 21]. Furthermore, evidence on the influence of age on the association of PF and AVF with systemic atherosclerosis is scarce [11]. Therefore, in the present study we investigated the association of PF and AVF with the severity of atherosclerosis in multiple arterial sites (aorta, coronary, carotid, and cerebral arteries) in a large autopsy study.

## Materials and methods

This study was conducted at the Sao Paulo Autopsy Service from University of Sao Paulo (Brazil). It was approved by the Ethics Committee in Research from University of Sao Paulo Medical School and complied with the 1975 Declaration of Helsinki. The deceased’s next of kin (NOK) was informed about this study, was invited to participate, and signed a written informed consent form. In the city of Sao Paulo, autopsy is compulsory for individuals whose cause of natural death is unclear [22]. Further details about Sao Paulo Autopsy Service and this study can be found elsewhere [23]. During 2011 to 2014, we included participants aged 30 years or above. The exclusion criteria were as follows: (1) the NOK provided inconsistent information during the clinical interview; (2) the NOK had less than weekly contact with the deceased; (3) the NOK was unable to participate due to emotional suffering; (4) subjects who had lost 10% or more of regular weight during the six months prior to death; (5) arteries or visceral fat was retained at autopsy by the pathologist; (6) subjects with post mortem interval ≥ 24 hours; and (7) subjects with signs of body autolysis according to the Crossley criteria [24].

### Clinical assessment

Information about the subject’s sociodemographic data (age, sex, race, years of education, marital status, and socioeconomic status [25]) and cardiovascular risk factors [hypertension, diabetes mellitus, dyslipidemia, coronary artery disease (CAD), heart failure, stroke, smoking, alcohol use, and physical inactivity] were collected from the deceased’s NOK through a semi-structured clinical interview [23, 26].

### Measurement of visceral fat

The heart together with the PF was washed in running water to remove clots; then, fixed in 70% alcohol by immersion for at least 24 hours, dissected, and weighed. Omental, mesenteric, mesocolon, and perirenal fat were dissected after the autopsy and weighed using a calibrated electronic scale. To avoid measurement error, we were especially careful to tare the scale before using it. The measurements were expressed in grams. AVF was determined by the sum of the omental, mesenteric, mesocolon, and perirenal fat.

### Atherosclerosis evaluation

We dissected the following:

1. The aorta from the ascending to the abdominal segment before the iliac bifurcation;
2. The common and internal carotid arteries;
3. The coronary arteries, including the left main, left anterior descending and right coronary artery as well as the circumflex artery; and
4. The cerebral arteries (e.g., basilar, posterior, posterior communicating, middle, anterior, anterior communicating, and internal carotid arteries proximal to the circle of Willis).

**Fig 1:**
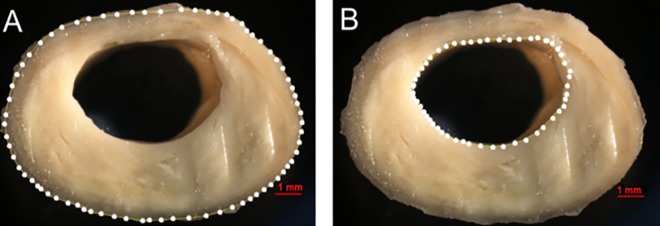
Calculation of the stenosis index in the carotid artery. (A) Area limited by the outer wall of the vessel. (B) Lumen area. A similar method was used to evaluate coronary and cerebral arteries.

All arteries were washed in running water to remove clots and fixed in 70% alcohol by immersion for 24 hours. Gelatin was injected inside the vessel lumen of the carotid, coronary, and cerebral arteries to prevent artery flatness. The arteries were then stored in 10% formalin. Subsequently, the carotid and coronary arteries were cut cross-sectionally at 5-mm intervals [27], and the cerebral arteries were cut at 3-mm intervals. We photographed the largest atheroma plaque in each artery using a stereomicroscope (Nikon® SMZ 1000, Nikon Inst., Tokyo, Japan). The areas delineated by the outer vessel wall and by the lumen were measured using the image software ImageJ® (Fig 1). The stenosis index was calculated by subtracting the lumen area from the outer area, dividing the difference by the outer area, and multiplying the result by 100 [28]. We used the mean stenosis index of all measured sections in each vessel bed (i.e., coronary, carotid, and cerebral arteries). We also counted the number of atherosclerotic plaques in the cerebral and coronary arteries as a measurement of atherosclerotic disease extent.

We assessed inter-rater reliability of stenosis index measurements in arterial segments. We randomly selected 164 segments, and two blinded independent raters measured the stenosis index. We calculated the intraclass correlation coefficient (ICC) using two-way mixed-effects model [29]. The inter-rater reliability was excellent with an ICC of 0.962 (95%CI = 0.948; 0.972).

**Fig 2:**
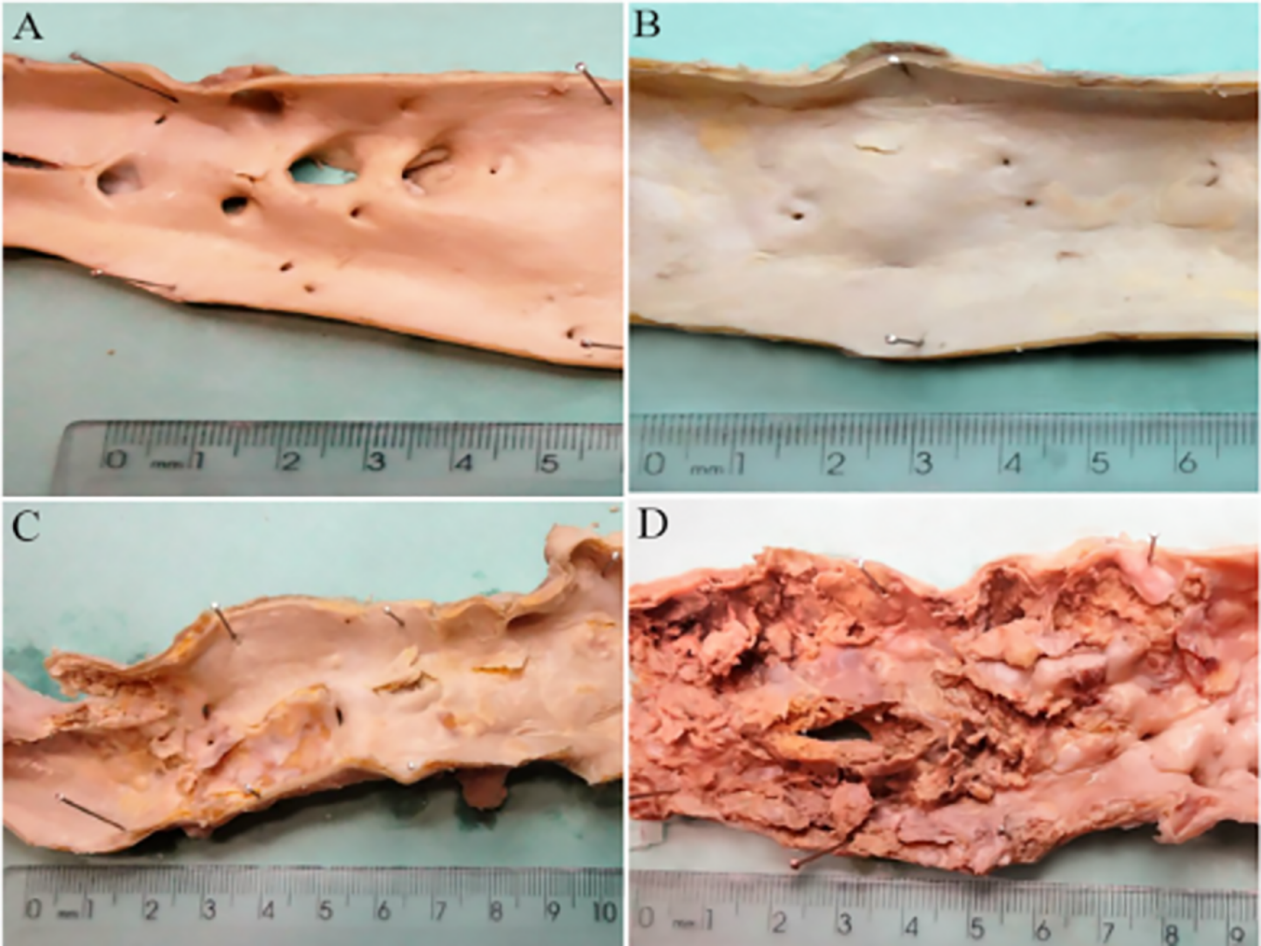
Evaluation of the severity of atherosclerosis in the aorta. (A) Absence of atherosclerosis. (B) Grade 1, non-confluent plaques without ulcerations and protrusions. (C) Grade 2, confluent areas or/and an area of ulceration with minimal protrusion. (D) Grade 3, confluent plaques with multifocal ulcerations or protrusions.

The aorta was opened longitudinally to investigate the severity of atherosclerosis and the presence of confluent lesions. Atherosclerosis in the aorta was classified as grade 1 (plaques were not confluent, and there were no ulcerations and protrusions); grade 2 (confluent areas or/and an area of ulceration with minimal protrusion); and grade 3 (confluent plaques, multifocal ulcerations, or protrusions) (Fig 2) [30].

## Statistical analysis

The sample size of 165 subjects was estimated based on previous studies [19] with a power of 90%, an alpha of 5%, and an effect size of 0.24 for the correlation between anterior epicardial fat surface and the score of coronary stenosis in a two sided-test. However, we opted to include 240 subjects to investigate the effect modification by age.

We defined the dependent variables as the stenosis indexes in carotid, coronary, and cerebral arteries (continuous variables); the number of atherosclerotic plaques in the coronary and cerebral arteries (discrete variables); and the severity of atherosclerosis in the aorta (ordinal variable). The independent variables were the PF and AVF weights (continuous variables). The sample characteristics were described with measurements of central tendency and dispersion for quantitative variables or proportions for qualitative variables.

The association of visceral fat with the stenosis indexes and the number of plaques in the coronary, carotid, and cerebral arteries was assessed using linear regression models. The association of visceral fat with the severity of atherosclerosis in the aorta was assessed using ordinal logistic regression. We adjusted all models for height [31–34], which was used as a measure of the participant’s size. We adjusted the multivariate models for age, sex, smoking status, alcohol use, physical inactivity, hypertension, and diabetes mellitus. We also evaluated the possibility of interaction [11] between age and visceral fat by creating an interaction term of age with AVF and PF and testing it in regression models in coronary, carotid and cerebral arteries adjusted for the same set of variables described above. The alpha level was set at 0.05 in two sided-tests. We used Stata/MP 13 (StataCorp LP, College Station, Texas, USA) for the statistical analyses.

## Results

**Fig 3:**
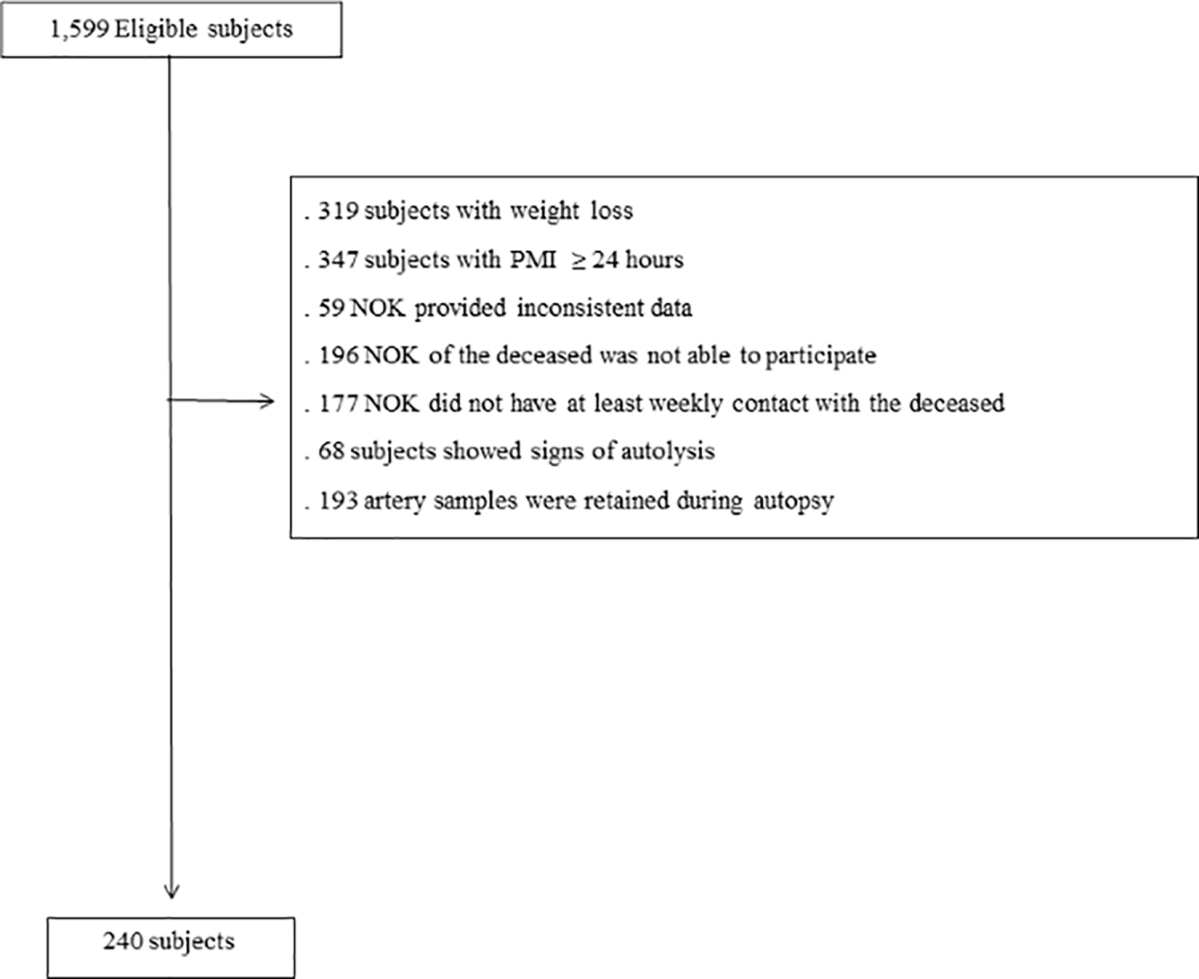
Flow chart of study population. NOK = next of kin; and PMI = post mortem interval.

Among 1,599 eligible subjects during the study period, 240 met the criteria for this study (Fig 3). The mean age of the subjects was 64.8±15.3 years (range = 30–98), 151 (63%) were male, 147 (61%) were White, and 197 (82%) of the NOK had daily contact with the deceased. The main cause of death was cardiovascular related 116 (48%) (Table 1). The mean weight of the AVF was 2,040±1,250 g, and the mean weight of the PF was 160±80 g. The mean stenosis index was 77.8±11.0% for coronary arteries, 64.1±0.4% for carotid arteries, and 52.8±0.5% for cerebral arteries. The mean number of plaques was 6.3±3.8 in coronary arteries; and 9.3±5.6 in cerebral arteries. Regarding the severity of aortic atherosclerosis, degrees 2 and 3 were most prevalent (40% each one), and no subject was devoid of atherosclerosis.

**Table 1:** Characteristics of the sample (n = 240).

Variable	Mean (standard deviation) or n (%)
Age (years)	64.8 (15.3)
Male	151 (62.9)
White	147 (61.2)
Married	122 (50.8)
Education (years), median (interquartile range)	4 (4)
Socioeconomic status (classes) ^a^	
. Upper	93 (38.8)
. Middle	129 (53.7)
. Lower	18 (7.5)
Daily contact of the next of kin with the deceased	197 (82.1)
Cardiovascular cause of death	116 (48.3)
Post mortem interval (hours)	14.7 (3.4)
Hypertension	163 (67.9)
Diabetes mellitus	67 (27.9)
Coronary artery disease	67 (27.9)
Heart failure	55 (22.9)
Dyslipidemia	36 (15.0)
Stroke	25 (10.4)
Smoking	
. Never	78 (32.5)
. Current	90 (37.5)
. Former	72 (30.0)
Alcohol use	
. Never	85 (35.4)
. Current	100 (41.7)
. Former	54 (22.5)
Physical inactivity	149 (62.1)

^a ^Socioeconomic status was defined according to gross family annual income in US$: Upper social class: ≥6,783; Middle: 3,256 to 6,782; Lower: ≤ 3,255 (1 dollar = 3.3 BRL)

### Association between visceral fat and aortic atherosclerosis

AVF was not associated with the severity of aortic atherosclerosis (p = 0.17), but greater PF was associated with the severity of aortic atherosclerosis in the multivariate analysis (coefficient = 4.39, 95%CI = 0.83; 7.94, p = 0.02) (Table 2).

**Table 2:** Association of visceral fat with the degree and extension of atherosclerosis (n = 240).

Arteries	Model 1^a^			Model 2^b^		
	Coef	95% CI	p	Coef	95% CI	p
**Stenosis index or degree of atherosclerosis**						
**Aorta**^**c**^						
AVF	0.20	0.001; 0.40	0.05	0.18	-0.07; 0.44	0.17
PF	5.65	2.61; 8.69	<0.0001	4.39	0.83; 7.94	0.02
**Coronary**^**d**^						
AVF	1.16	-0.01; 2.33	0.05	1.49	0.15; 2.83	0.03
PF	21.69	4.92; 38.47	0.01	17.07	-0.85; 34.99	0.06
**Carotid**^**d**^						
AVF	-0.10	-0.88; 0.68	0.81	-0.23	-1.15; 0.68	0.63
PF	0.60	-11.15; 12.35	0.92	-4.13	-16.78; 8.51	0.52
**Cerebral**^**d**^						
AVF	0.38	-0.51; 1.27	0.40	0.32	-0.69; 1.34	0.53
PF	4.90	-7.99; 17.80	0.45	1.49	-12.08; 15.07	0.83
**Number of plaques**						
**Coronary**^**d**^						
AVF	0.69	0.28; 1.10	0.001	0.71	0.24; 1.19	0.003
PF	8.54	2.60; 14.48	0.005	6.25	-0.13; 12.63	0.05
**Cerebral**^**d**^						
AVF	0.48	-0.13; 1.10	0.12	0.41	-0.26; 1.08	0.23
PF	7.06	-1.69; 15.81	0.11	0.96	-7.96; 9.88	0.83

Coef = coefficient; CI = confidence interval; AVF = abdominal visceral fat; PF = Pericardial fat

^a^ Model 1: Adjusted for height

^b^ Model 2: Adjusted for height, age, sex, smoking, alcohol use, physical inactivity, hypertension, and diabetes mellitus

^c^ Ordered logistic regression

^d ^Linear regression

### Association between visceral fat and coronary artery atherosclerosis

**Fig 4:**
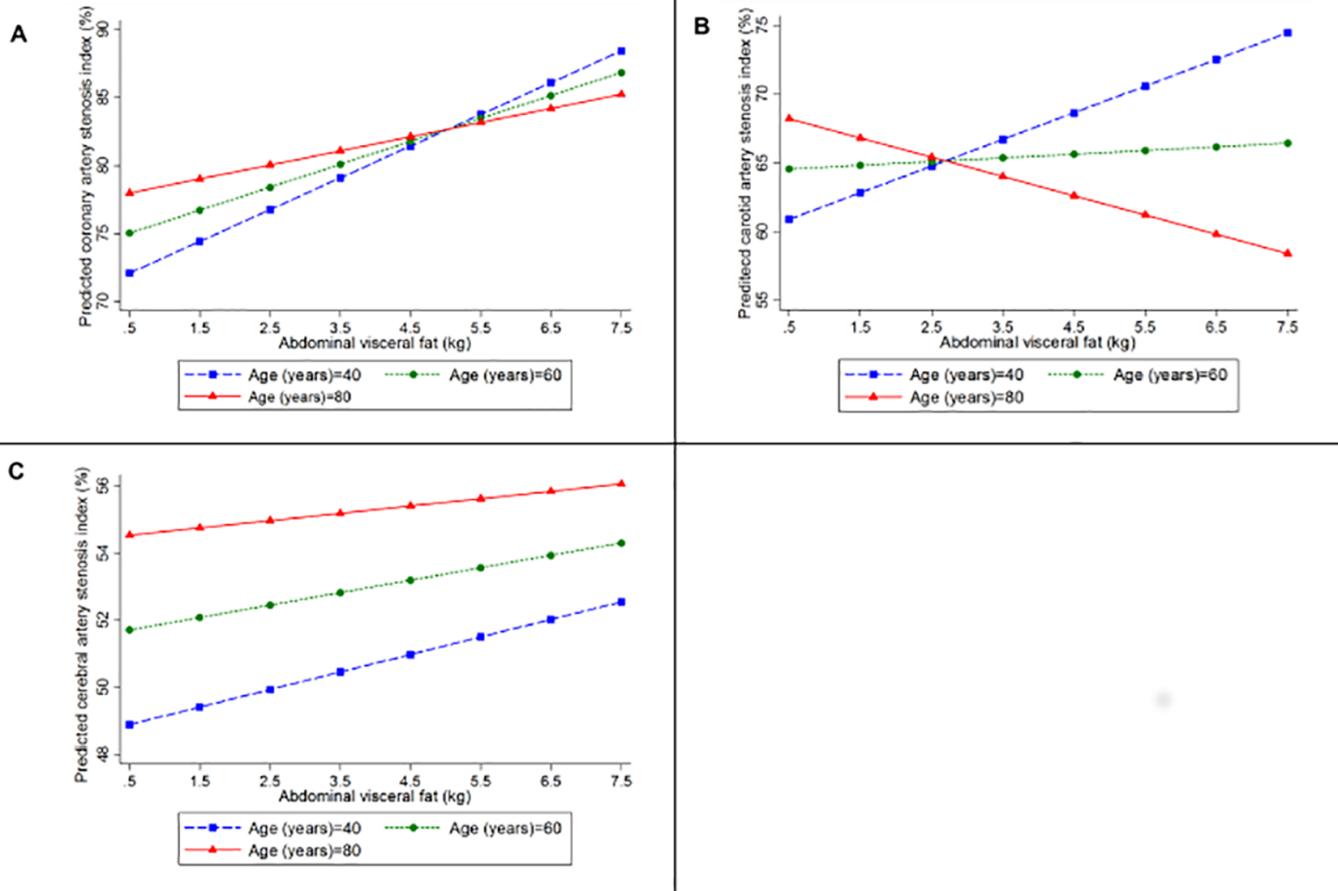
**Predicted values of the stenosis index in (A) coronary, (B) carotid, and (C) cerebral arteries, according to the amount of abdominal visceral fat calculated for participants with 40 (blue line), 60 (green line), and 80 (red line) years old, using linear regression models adjusted for height, age, sex, smoking status, alcohol use, physical inactivity, hypertension, and diabetes mellitus, and including an interaction between age and abdominal visceral fat**.

**Fig 5:**
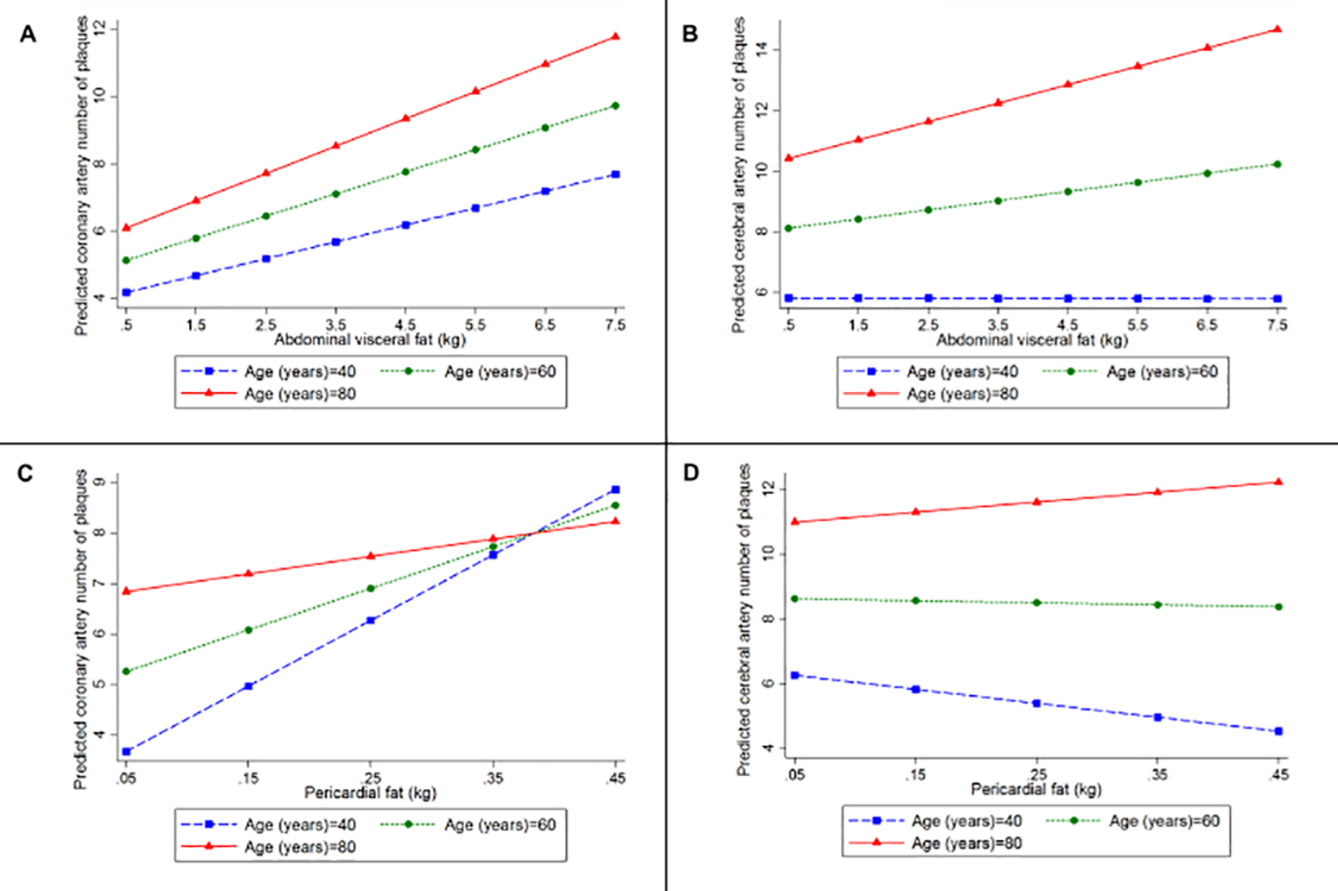
**Predicted values of the number of plaques in (A) coronary and (B) cerebral arteries according to the amount of abdominal visceral fat, and in the (C) coronary and (D) cerebral arteries according to the amount of pericardial fat for participants with 40 (blue line), 60 (green line), and 80 (red line) years old, using linear regression models adjusted for height, age, sex, smoking status, alcohol use, physical inactivity, hypertension, and diabetes mellitus, and including an interaction between age and abdominal visceral fat or pericardial fat**.

**Fig 6:**
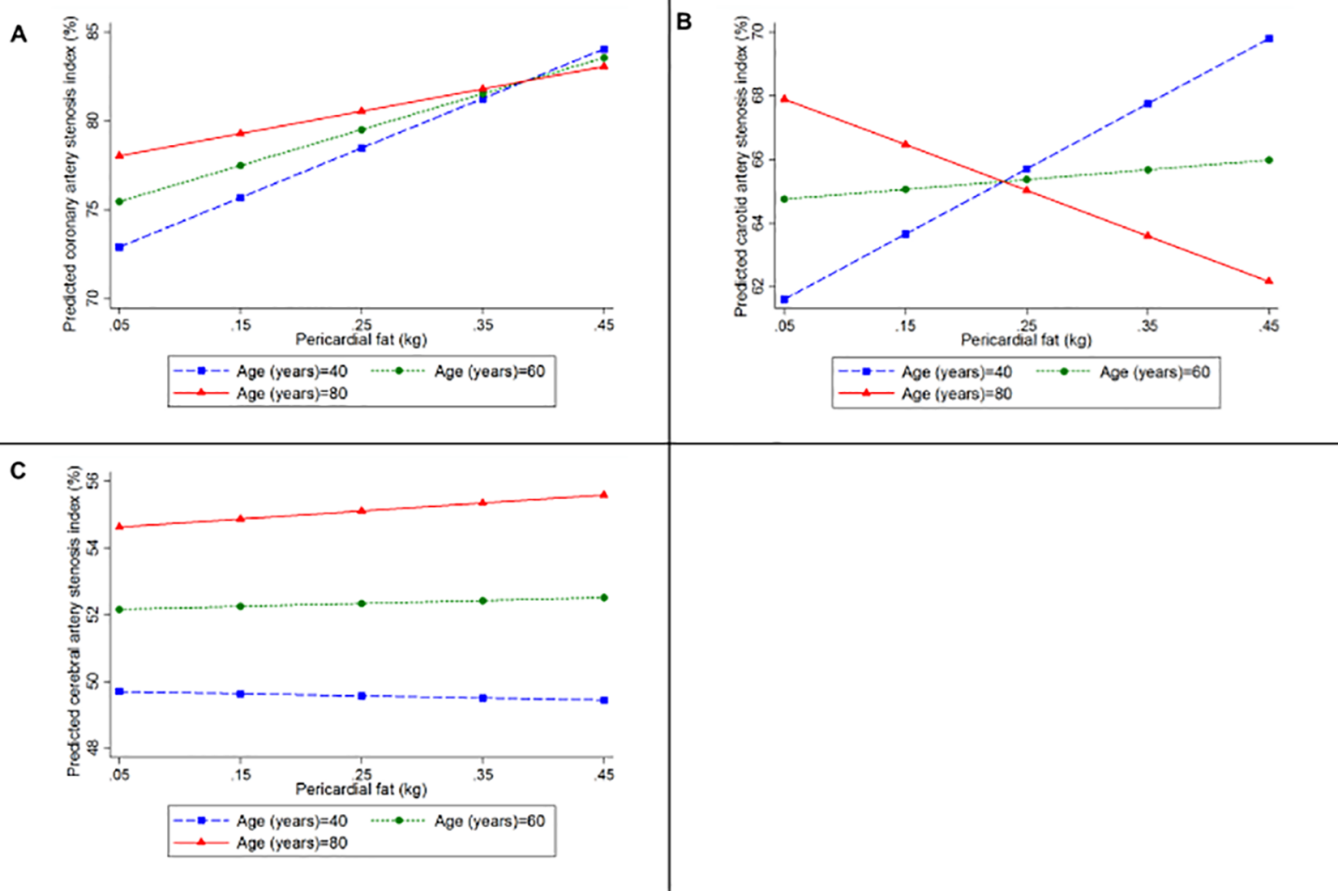
**Predicted values of the stenosis index in (A) coronary, (B) carotid, and (C) cerebral arteries, according to the amount of pericardial fat calculated for participants with 40 (blue line), 60 (green line), and 80 (red line) years old, using linear regression models adjusted for height, age, sex, smoking status, alcohol use, physical inactivity, hypertension, and diabetes mellitus, and including an interaction between age and pericardial fat**.

A greater amount of AVF was associated with a higher stenosis index (coefficient = 1.49, 95%CI = 0.15; 2.83, p = 0.03) and a greater number of plaques in coronary arteries (coefficient = 0.71, 95%CI = 0.24; 1.19, p = 0.003) in the multivariate analyses (Table 2). However, there was no interaction of AVF and age regarding the atherosclerotic burden (p = 0.47) (Fig 4A), nor on the extent of atherosclerosis (p = 0.68) in coronary arteries (Fig 5A) (Table 3). Despite the lack of statistical significance, a trend was noted for the association of PF with coronary atherosclerosis as measured by the stenosis index (p = 0.06) and with the number of atherosclerotic plaques (p = 0.05) (Table 2). We did not observe any interaction between PF and age on coronary atherosclerotic burden (p = 0.53) (Fig 6A), nor on its extent (p = 0.28) (Fig 5C) (Table 3).

**Table 3:** Association between visceral fat and atherosclerosis in different arterial sites, considering an interaction term between visceral fat and age (n = 240).

Arteries	Model 1^a^			Model 2^b^		
	Coef	95% CI	p	Coef	95% CI	p
**Stenosis index**						
**Coronary**						
AVF	5.03	-0.42; 10.48	0.07	3.63	-2.35; 9.60	0.23
Age	0.25	0.08; 0.43	0.005	0.16	-0.03; 0.36	0.10
AVF * age	-0.06	-0.14; 0.02	0.13	-0.03	-0.12; 0.05	0.47
PF	52.64	-26.00; 131.29	0.19	43.18	-40.15; 126.52	0.31
Age	0.21	0.01; 0.40	0.03	0.15	-0.06; 0.35	0.16
PF * age	-0.54	-1.68; 0.60	0.35	-0.38	-1.58; 0.81	0.53
**Carotid**						
AVF	5.30	- 1.55; 9.05	0.01	5.29	1.12; 9.45	0.01
Age	0.24	0.12; 0.36	<0.0001	0.22	0.09; 0.36	0.001
AVF * age	-0.08	-0.14; -0.03	0.003	-0.08	-0.14; -0.02	0.009
PF	54.14	-0.44; 108.71	0.05	55.33	-2.92; 113.59	0.06
Age	0.22	0.08; 0.35	0.002	0.20	0.06; 0.34	0.01
PF * age	-0.85	-1.64; -0.06	0.03	-0.87	-1.70; -0.04	0.04
**Cerebral**						
AVF	0.47	-3.65; 4.59	0.82	0.83	-3.71; 5.36	0.72
Age	0.16	0.03; 0.29	0.01	0.14	-0.003; 0.29	0.06
AVF * age	-0.005	-0.07; 0.05	0.85	-0.01	-0.07; 0.06	0.82
PF	-17.46	-76.79; 41.87	0.56	-3.67	-66.78; 59.43	0.91
Age	0.12	-0.02; 0.26	0.10	0.12	-0.03; 0.27	0.13
PF * age	0.24	-0.62; 1.10	0.58	0.07	-0.83; 0.98	0.87
**Number of plaques**						
**Coronary**						
AVF	0.85	-1.18; 2.88	0.41	0.19	-2.09; 2.48	0.87
Age	0.07	0.004; 0.13	0.04	0.04	-0.03; 0.11	0.23
AVF * age	-0.004	-0.03; 0.02	0.79	0.01	-0.03; 0.04	0.68
PF	21.94	-5.90; 49.77	0.12	22.49	-7.63; 52.62	0.14
Age	0.09	0.02; 0.16	0.01	0.09	0.02; 0.17	0.02
PF * age	-0.23	-0.63; 0.17	0.26	-0.24	-0.67; 0.19	0.28
**Cerebral**						
AVF	-0.50	-3.35; 2.34	0.73	-0.61	-3.81; 2.58	0.70
Age	0.14	0.05; 0.23	0.001	0.11	0.01; 0.21	0.03
AVF * age	0.01	-0.03; 0.05	0.64	0.01	-0.03; 0.06	0.52
PF	-11.02	-49.36; 27.31	0.57	-11.70	-53.02; 29.62	0.58
Age	0.13	0.04; 0.23	0.005	0.11	0.01; 0.21	0.04
PF * age	0.17	-0.38; 0.73	0.53	0.18	-0.40; 0.77	0.54

Coef = coefficient; CI = confidence interval; AVF = abdominal visceral fat; PF = Pericardial fat

^a^Model 1: Linear regression model, adjusted for height and age, including an interaction term between visceral fat and age

^b^Model 2: Linear regression model, adjusted for height, age, sex, smoking status, alcohol use, physical inactivity, hypertension, and diabetes mellitus, including an interaction term between visceral fat and age

### Association between visceral fat and carotid artery atherosclerosis

AVF (p = 0.63) and PF (p = 0.52) were not associated with the carotid artery stenosis index (Table 2) in multivariate analysis. However, we observed an interaction of age with both AVF (coefficient = -0.08; 95%CI = -0.14; -0.02, p = 0.009) (Fig 4B) and PF (coefficient = -0.87; 95%CI = -1.70; -0.04, p = 0.04) (Fig 6B) (Table 3). While middle-aged adults showed a worse atherosclerotic burden in carotid arteries with increases in AVF and PF, we observed an inverse association of carotid artery atherosclerotic burden with the AVF and PF weight in the oldest subjects.

### Association between visceral fat and cerebral artery atherosclerosis

AVF was not associated with the cerebral artery stenosis index (p = 0.53) nor with the number of cerebral artery atherosclerotic plaques after adjusting for confounding factors (p = 0.23) (Table 2). We did not observe an interaction between AVF and age on cerebral artery atherosclerotic burden (p = 0.82) (Fig 4C) nor on its disease extent (p = 0.52) (Fig 5B) (Table 3). Similarly, multivariate analysis showed that PF was also not associated with the stenosis index in cerebral arteries (p = 0.83) nor with the number of plaques (p = 0.83) (Table 2). The interaction between age and PF on atherosclerotic burden (p = 0.87) (Fig 6C) and on its extent (p = 0.54) (Fig 5D) was not significant (Table 3).

## Discussion

Our study has demonstrated that the association between visceral fat and atherosclerosis is highly variable depending on the location of visceral fat and the vascular bed. While PF was associated with atherosclerotic burden in the aorta and marginally associated with coronary artery, AVF was associated with coronary artery atherosclerosis. On the other hand, visceral fat was not associated with atherosclerosis in the cerebral and carotid arteries. Interestingly, the effect of visceral fat on carotid artery atherosclerotic burden seems to be modified by age.

Visceral fat seems to have local and systemic effects on atherosclerosis pathophysiology [35–37]. Among the systemic inflammatory effects, macrophage infiltration was found in the AVF in obese individuals [35]. These cells are involved in the production of adipokines, which are related to metabolic syndrome [38], and increased cardiovascular risk [39]. Previous imaging studies have shown that larger deposits of AVF were associated with higher calcification scores in the abdominal aorta [40, 41], which contradicts our current findings. However, some important methodological differences need to be highlighted. First, we examined the whole aorta; and second, we evaluated the confluence, ulceration, and protrusion of the plaques, including non-calcified plaque components that are not represented by the calcium score [30]. On the other hand, we found that AVF was indeed associated with the burden and extent of coronary artery atherosclerosis, which corroborates previous imaging studies [9, 42]. However, these studies did not evaluate the atherosclerosis burden as a continuous variable, but as a categorical variable. Kortelainen and Sarkioja were the first to use autopsy material, and they also found an association between AVF and coronary narrowing [7, 8]. However, in one study [20], they did not find an association between AVF and the extent of coronary atherosclerosis; and in another study, they did not evaluate disease extent [7]. Moreover, their samples were restricted to middle-age White subjects [7, 8, 20]. Our results extend this knowledge to a larger and ethnically diverse sample with a wider age range. Interestingly, no association was noted between AVF and carotid or cerebral artery atherosclerosis. This finding contrasts with previous imaging-based studies, which found an association between AVF and CIMT [11], as well as with total cerebral artery plaque volume; however, that study was limited by a small sample size of 25 subjects, which only allowed for univariate analysis [14]. In another study, AVF was associated with stenosis or occlusion in cerebral arteries; however, stenosis was evaluated as a categorical variable using the cut-off of ≥70%, and the participants were Asian and aged 40 years or older [43].

On the other hand, the effect of visceral fat on atherosclerosis may not be fully explained by systemic effects. In fact, prior data suggest that epicardial fat can locally contribute to atherosclerosis by lipotoxicity, cytokine secretion, and the increased production of hemostatic factors [44], thereby inducing an inflammatory response that may play a role in coronary atherogenesis [37, 45]. Previous studies have already demonstrated that the effect of epicardial fat may extend to aortic atherosclerosis as measured by the percentage of obstruction by computed tomography [13]. In our study, we observed an association between PF and the severity of aorta atherosclerosis. However, PF was marginally associated with the burden and extent of coronary artery atherosclerosis. Most of the previous imaging studies evaluated only the epicardial fat [19, 46] or the epicardial and paracardial fat separately [18], and few studies evaluated the PF itself [5, 6]. Moreover, epicardial and paracardial fat were estimated indirectly using computed tomography [5, 6, 18, 46] or computerized photographs of the heart to quantify the epicardial fat thickness and area [19]. In addition, analyses were adjusted only for body mass index [18] or age [7] in some studies, while we adjusted our regression models for a more comprehensive set of possible confounding factors. Moreover, coronary artery atherosclerosis was evaluated macroscopically in some autopsy studies [18, 19], instead of using more robust morphometric measurements such as the ones that we performed. Finally, differences in age and race composition could also explain the marginal association between PF and coronary artery atherosclerosis in our study. Since we compared AVF with PF in the same individuals under the same conditions, and adjusting for the same set of confounding factors, our results suggest AVF seems to have more effect in coronary artery atherosclerosis than PF. Therefore, coronary artery atherosclerosis may be more influenced by systemic than local effects, while aorta seems to be more influenced by local effects. We did not find an association between PF and carotid or cerebral artery atherosclerosis. In contrast, previous studies have found an association between CIMT and epicardial fat [12, 15], but data regarding cerebral arteries were not found in the literature.

We were also able to demonstrate the effect modification of age on both the association of AVF and PF with carotid artery atherosclerosis. While we found a positive association of visceral fat with carotid artery atherosclerosis in younger individuals, this association became negative in older adults. Although this might be related to survival bias, one may speculate whether the effect might be variable across different age groups. Greater AVF and PF were related to higher coronary atherosclerosis burden [5–9, 18–20, 46]. Nevertheless, since CAD may develop before carotid atherosclerosis [47], individuals with greater AVF and PF may die earlier from CAD, leading to a reverse association between visceral fat and carotid atherosclerosis in older individuals; such a phenomenon is known as the “obesity paradox” that has been described in other chronic diseases [48]. However, longitudinal studies with multiple measurements of visceral fat and carotid artery atherosclerosis across the lifespan would be necessary to confirm this finding. We did not observe an interaction of age with AVF and PF regarding atherosclerosis in the coronary and cerebral arteries. To our knowledge, these interactions were not evaluated in previous studies.

The results of our study should be interpreted with consideration of some limitations. First, since this was a cross-sectional study, we could not establish the causal relationship between visceral adiposity and systemic atherosclerosis. Moreover, information about cardiovascular risk factors was collected post mortem. Previous studies from our group showed good reliability of the data collected from the NOK [26, 49], and we excluded participants with limited contact with the NOK. Additionally, it is important to highlight that the main measurements (visceral fat and atherosclerosis) were not affected by the lack of follow-up since they were measured directly. On the other hand, our study has several strengths. We used morphometric measurements of atherosclerosis rather than using estimated atherosclerosis quantification [7, 8, 18, 19]. Similarly, we used direct measurements to quantify the visceral fat instead of imaging methods [5, 6, 12–15, 18, 19, 46]. Another strength was the larger sample size compared to previous autopsy studies that assessed a maximum of 116 individuals [7, 8, 18–20], allowing us to test the interaction between age and visceral fat. Furthermore, the mean post mortem interval of the participants was short compared with other studies [19] in that the autopsy occurred within 48 hours after death. Finally, to the best of our knowledge, this is the first autopsy study to investigate the association of AVF and PF with systemic atherosclerosis in addition to coronary artery atherosclerosis. Future research should be directed towards the assessment of the atherosclerotic plaque composition and the fibrous cap thickness.

## Conclusion

We found that AVF was associated with coronary artery atherosclerosis. Only PF was related to the severity of atherosclerosis in the aorta and marginally associated with coronary atherosclerosis. We also found an interaction of age with AVF and PF regarding the carotid artery stenosis index. Understanding the association between specific visceral fat deposits and systemic atherosclerosis is important for the identification of individuals at higher risk in order to promote preventive actions and reduce atherosclerotic events.
